# Chronic LCMV Infection Is Fortified with Versatile Tactics to Suppress Host T Cell Immunity and Establish Viral Persistence

**DOI:** 10.3390/v13101951

**Published:** 2021-09-29

**Authors:** Caleb J. Studstill, Bumsuk Hahm

**Affiliations:** Departments of Surgery and Molecular Microbiology & Immunology, University of Missouri-Columbia, Medical Science Building M331, One Hospital Drive, Columbia, MO 65212, USA; calebjstudstill@gmail.com

**Keywords:** LCMV, persistent viral infections, viral immunology, immunology models, T cell immunology, T cell exhaustion

## Abstract

Ever since the immune regulatory strains of lymphocytic choriomeningitis virus (LCMV), such as Clone 13, were isolated, LCMV infection of mice has served as a valuable model for the mechanistic study of viral immune suppression and virus persistence. The exhaustion of virus-specific T cells was demonstrated during LCMV infection, and the underlying mechanisms have been extensively investigated using LCMV infection in mouse models. In particular, the mechanism for gradual CD8^+^ T cell exhaustion at molecular and transcriptional levels has been investigated. These studies revealed crucial roles for inhibitory receptors, surface markers, regulatory cytokines, and transcription factors, including PD-1, PSGL-1, CXCR5, and TOX in the regulation of T cells. However, the action mode for CD4^+^ T cell suppression is largely unknown. Recently, sphingosine kinase 2 was proven to specifically repress CD4^+^ T cell proliferation and lead to LCMV persistence. As CD4^+^ T cell regulation was also known to be important for viral persistence, research to uncover the mechanism for CD4^+^ T cell repression could help us better understand how viruses launch and prolong their persistence. This review summarizes discoveries derived from the study of LCMV in regard to the mechanisms for T cell suppression and approaches for the termination of viral persistence with special emphasis on CD8^+^ T cells.

## 1. Introduction

The lymphocytic choriomeningitis virus (LCMV) system is one of the most widely used infection models for the study of virus-host immunity interactions. In large part, this feat was possible due to the virus’s natural host, the mouse, which is useful for studying biological concepts at the organismal level. The virus has proven easy to manipulate in cell culture, and it is used as a model for more pathogenic arenaviruses (e.g., Lassa virus) and to study meningitis diseases. In particular, the potent antiviral T cell response generated against prototypic LCMV strains has become a characteristic model for host immunity to acute and chronic viral infections. As a result, many seminal findings have been achieved due to LCMV research, including insights into major histocompatibility restriction and T cell memory. Moreover, the complex host-virus interactions leading to an establishment of a persistent viral infection by other LCMV strains has fascinated many researchers and led to the discovery of T cell exhaustion. This finding has been extended to other chronic virus infections in humans as well as cancer studies and significantly influenced the development of current PD-1-targeted cancer immunotherapies. This review will place a focus on the mechanisms for immune suppression caused by chronic LCMV infections. 

## 2. LCMV Clone 13 and Viral Persistence

Work with LCMV culminated in several key studies that identified LCMV variants capable of causing persistent viral infection [1,2,3]. Of these, LCMV clone 13 (Cl 13), which was isolated from the spleens of mice infected neonatally with LCMV Armstrong strain clone 53b (Arm), was shown to downregulate antiviral T cell responses and was able to remain at high titers for long periods of time in mice [2]. Thus, LCMV Cl 13 became a suitable model for the study of chronic viral infections. Adding to the complexity of how LCMV Cl 13 establishes persistent infection, LCMV Cl 13 differs from LCMV Arm by 5 nucleotides. Only two of these nucleotide changes result in fundamental changes in the amino acid sequence, one in glycoprotein (GP)-1 and the other in the L polymerase [4]. Specifically, a change in phenylalanine to leucine at position 260 in GP-1 was shown to be critical for the generation of a viscerotropic LCMV strain (persistent) [5,6]. This substitution led to better infectivity of macrophages and dendritic cells (DCs), including plasmacytoid dendritic cells, due to enhanced binding with the alpha-dystroglycan receptor [7,8]. These cells, especially DCs, express higher levels of alpha-dystroglycan, which makes them important in the course of LCMV Cl 13 infection. Additionally, the change in L, lysine to glutamine at residue 1079 leads to increased viral replication within infected cells (e.g., macrophages) [4,7]. Since the two variants are so similar, T cell epitopes recognized against each virus are the same. Thus, minor variations in the viral genome can lead to very significant changes in how the virus interacts with the host. 

## 3. CD8^+^ T Cell Exhaustion

Early work with the persistent LCMV variants showed that the cytotoxic lymphocyte response in chronically infected mice (carrier mice) was suppressed when compared to mice infected with LCMV Arm [2,9,10]. However, the mechanism of LCMV Cl 13 persistence had not been documented. It was later elucidated that the LCMV Cl 13 strain eventually induces an absence of antiviral CD8^+^ T cell responses, which prevents clearance of the virus [11]. Thus, the term “exhaustion” in the context of antiviral T cell responses was introduced. This was clarified to show that the deletion of antiviral cytotoxic T cells was specific to the NP397 epitope-recognizing CD8^+^ T cells, and the GP33 epitope-recognizing effector CD8^+^ T cells were not completely deleted but were unable to elicit an antiviral immune response [12]. Subsequent studies have focused many efforts at defining and elucidating the mechanisms of T cell exhaustion. 

The definition of an exhausted T cell has evolved. Initially, exhausted CD8^+^ T cells were described as being absent or lacking cytotoxic and antiviral functions [11,12]. This was later expanded to include a progressive loss of the capacity to produce antiviral cytokines such as IL-2, tumor necrosis factor alpha (TNFα), and interferon γ (IFNγ), cells’ inability to proliferate in response to antigenic stimulation, and eventual deletion of the cell itself through mechanisms dependent on Fas/Fas ligand (FasL), perforin, or the TNF receptor (TNFR) [13,14]. The exhaustion process also has severe impacts on the generation of memory CD8^+^ T cell phenotypes [15,16]. Further genomic analysis of exhausted CD8^+^ T cells and the identification of surface inhibitory markers (e.g., programmed cell death protein 1, PD-1) on exhausted cells revealed a more nuanced picture in that exhausted cells reflect a phenotype not wholly representative of other known T cell phenotypes, including naïve, effector, memory, or anergy states [17,18]. In addition, exhausted CD8^+^ T cells have altered metabolic and bioenergetic pathways, and downregulated translational abilities [18,19]. 

More recent analyses have added further depth to the definition of exhausted CD8^+^ T cells, indicating that the progressive tendencies of the exhaustion phenotype give rise to a heterogeneous population of exhausted cells as the infection continues. Initially, two subpopulations of exhausted CD8^+^ T cells were identified: a memory-like population consisting of T cell factor 1 (TCF1)^hi^ T-box expressed in T cells (T-bet)^hi^ PD-1^lo^ cells, which could potentially be revitalized with the use of inhibitory receptor blockade, and a terminally exhausted population consisting of TCF1^neg^ PD-1^hi^ eomesodermin (Eomes)^hi^ CD39^hi^ cells [20,21]. These populations have also been suggested to be dependent on tissue localization. For example, it was noted that later during chronic infection a larger portion of the terminally exhausted T cells were located in the peripheral tissues [13,22]. 

As the memory-like subset may provide clues to the regulation of the exhaustion state, further studies sought to investigate these cells in more detail. This subset was found to be T follicular helper (Tfh)-like CD8^+^ T cells responsible for the so-called “proliferative burst” following checkpoint blockade [20,23,24,25,26]. These cells express TCF1, C-X-C chemokine receptor type 5 (CXCR5), and PD-1 but not T-cell immunoglobulin and mucin-domain containing-3 (Tim-3) (TCF1^hi^PD-1^+^CXCR5^+^Tim3^−^), and regulation of this cell type was dependent on B lymphocyte induced maturation protein 1 (Blimp-1), DNA-binding protein inhibitor ID-2 (ID2), E2A, and B-cell lymphoma 6 protein homolog (Bcl6) signaling. These cells were also suggested to be vital for clearing viral infections from lymphoid tissues (particularly B cell follicles) as they had an increased ability to reduce viral loads and worked synergistically with treatments targeting PD-1 and PD-1 ligand-1 (PD-L1). 

Another group of transitional, less exhausted cells, residing between the memory-like cells and terminally exhausted cells, was identified based on the expression of the glycoprotein CD101 [27]. The progenitor or memory-like TCF1^hi^ population of exhausted CD8^+^ T cells was shown to differentiate first into CD101^−^Tim3^+^ cells, which exhibited reduced TCF1 expression and an effector-like transcriptional profile, including the expression of CX3C chemokine receptor 1 (CX3CR1), production of pro-inflammatory cytokines and granzyme B. In addition, these cells contributed to viral control [27,28]. These cells eventually convert into CD101^+^Tim3^+^ CD8^+^ T cells, representing a more exhausted phenotype. Independent observations by another team identified a similar group of effector-like exhausted cells that express CX3CR1 and have increased cytolytic abilities, which makes them critical for viral control [29]. This study further defined the subsets of exhausted CD8^+^ T cells into three populations: (1) a memory-like, progenitor cell subset that expresses TCF1, Ly108, CXCR5, and PD-1, does not express Tim-3 or CD101, and had the most self-renewing properties; (2) an effector-exhausted subset that expresses CX3CR1, PD-1, and Tim-3, were CD101-negative and TCF1-negative, and display some cytotoxic abilities; (3) a terminally exhausted subset that expresses PD-1, Tim-3, and CD101, does not express CX3CR1 or Ly108, and are highly dysfunctional [29]. All three subtypes display differential transcriptional profiles, phenotypical properties, functionality, and tissue localization. Essentially, this scheme reciprocates the progressive exhaustion phenotype hypothesis in that progenitor cells give rise to effector exhausted or terminally exhausted cells, and effector exhausted cells can give rise to terminally exhausted cells. It should be noted that a fourth population of exhausted CD8^+^ T cells was identified based on single-cell RNA sequencing analysis and termed “proliferating” due to the mRNA expression profile of *Mki67* (Ki67) and *Top2a*, which appeared to represent *Havcr2* (Tim-3), *Pdcd1* (PD-1), and *Cx3cr1*-expressing cells that do not express *Tcf7* (TCF-1), *Slamf6* (Ly108), or *Cd101* and have a phenotype more similar to terminally exhausted cells than to stem-like cells [30]. However, these observations were based on transcriptional profiling. The role of these cells during LCMV Cl 13 infection and whether these cells represent another transitory population within the effector-exhausted subpopulation or simply proliferating versions of effector-exhausted CD8^+^ T cells is unknown. 

A recent study sought to gain a better understanding of the role of these exhausted CD8^+^ T cell subsets and their distribution within infected mice. Utilizing transcriptional profiles from the above studies, the authors identified five functional groups of exhausted CD8^+^ T cells: (1) memory (stem)-like, (2) proliferating, (3) effector-like, (4) intermediate exhaustion, and (5) advanced exhaustion [31]. The stem-like population, exhibiting characteristics as defined above, was primarily found in the secondary lymphoid organs (spleen and lymph nodes). The proliferating population was found in the lymphoid and peripheral tissues at low frequencies. The effector-like population had high *Cx3cr1* and *Gzmb* (granzyme B) expression, expressed several molecules important for lymphocyte trafficking (e.g., *S1pr1,* sphingosine 1-phosphate receptor 1), and were found mostly in the lung, blood, and spleen. The intermediate exhausted population was found in all tissues and displayed a transitory phenotype between effector-like and terminally exhausted. Finally, the advanced (terminally) exhausted population was found in all tissues and made up the major populations of cells in the liver and bone marrow. This population generally had higher levels of inhibitory gene expression (e.g., *Pdcd1*, *Cd160*, *Lag3*), and lower levels of molecules associated with T cell receptor (TCR) activation (*Nfkbid*, *Jun*, *Jund*). Interestingly, the authors found that the different populations generally displayed plasticity in their function. To show this, exhausted CD8^+^ T cells from specific tissues were taken and transferred into infection-matched recipient mice (at the same point of infection, i.e., 14 days post infection). Transferred cells did not home preferentially to their originating tissue, but they were able to change their phenotype to more closely resemble their new tissue of residency. However, this was not the case for cells from the liver, which had more advanced exhausted cells. While most cells did transition to the phenotype associated with their new tissue localization, there seems to be some transitory lag as certain populations retained characteristics of their original phenotype. Also, plasticity was much less prevalent later on in the progression of the chronic infection (i.e., 21 days post infection). This work demonstrates that the exhausted CD8^+^ T cell population is extremely heterogeneous, and the development of exhaustion is dependent on the environment in which the T cells reside. Furthermore, these studies highlight the ability of the populations to remain “plastic” in that certain phenotypes are not as terminal as previously thought.

## 4. CD4^+^ T Cell Exhaustion

Many T cell exhaustion studies have primarily focused on CD8^+^ T cells. However, it was appreciated from early experiments how important CD4^+^ T cell help was to the eventual resolution of LCMV Cl 13 infection and in the maintenance of antiviral CD8^+^ T cell responses throughout the exhaustion phase [12,32,33,34]. For instance, the transfer of unexhausted CD4^+^ T cells into a mouse infected with LCMV Cl 13 greatly improves the functionality of exhausted CD8^+^ T cells and promotes viral clearance [35]. Virus-specific CD4^+^ T cells have also been shown to display an exhausted phenotype similar to exhausted, CD8^+^ T cells, which appears to occur early during LCMV Cl 13 infection [14,36,37]. Heterogeneity in the exhausted CD4^+^ T cell population has been observed as some subsets of CD4^+^ T cells are able to persist throughout infection while others are functionally diminished early in the course of infection [36]. Furthermore, it appears that CD4^+^ T cell exhaustion is not merely a loss of function but a more nuanced altered functionality as some CD4^+^ T cells retain their ability to produce antiviral cytokines [36,38]. One explanation for this is that viral persistence may push CD4^+^ T cells from a primarily antiviral, helper T cell 1 (Th1) phenotype to a Tfh cell phenotype [39]. This may be counterproductive for the present viral infection but may aid in the eventual clearance of the virus. Exhausted CD4^+^ T cells have distinct transcriptional profiles from effector and memory CD4^+^ T cells, and exhausted CD8^+^ T cells, though some similar pathways between exhausted T cells exist [38]. Therefore, T cell exhaustion is an intricate phenotype in that both CD8^+^ T cells and CD4^+^ T cells can become exhausted, but this seemingly occurs by different mechanisms.

Unlike acute infection, strong TCR signaling induces the development of Tfh cells and the strength of the signal inversely correlates with Th1 differentiation during chronic LCMV infection [40]. Linking TCR signal to enhanced Tfh differentiation during chronic LCMV infection was partly supported by an earlier study on the role of the signaling adaptor CD2-associated protein (CD2AP). Inactivation of CD2AP was shown to promote CD4^+^ Tfh cell differentiation and germinal center response, leading to enhanced control of viral infection [41]. 

Previously, CD30, a member of the TNFR superfamily, was reported to regulate multiple CD4^+^ Tfh cell responses, such as providing help for memory B cell responses [42]. However, during persistent infection with LCMV Cl 13 the level of T cell exhaustion or viral control did not change in CD30-deficient mice compared to WT mice, indicating that CD30 was proven to have no clear role in CD4^+^ or CD8^+^ T cell responses [43]. 

## 5. Regulation of T Cell Exhaustion

The initiation of T cell exhaustion is a complex process requiring many interlinking factors. Studies with LCMV Cl 13 have discovered several factors that may be primarily involved in this process. The creation of a T cell-intrinsic or extrinsic suppressive environment is due to high antigen levels, disruption of antigen presentation, upregulation of inhibitory receptors, synthesis of immunoregulatory cytokines, and changes within the epigenetic and transcriptional regulation of T cells. Several of these factors as they pertain to LCMV Cl 13 are discussed below. 

### 5.1. High Antigen Burden

Early work with some LCMV strains suggested that persistence was due to rapid dissemination of the virus throughout the tissues [44]. This systemic spread was observed with LCMV Cl 13, and it was suggested that high viral titers, and in correlation high antigen loads, contribute to the downregulation of antiviral T cell responses [2,45]. Later use of a perforin-deficient mouse model, which is incapable of clearing the virus, added more substance to this hypothesis [14]. In this study, high viral loads led to a reduction in antiviral CD8^+^ T cell effector functions as well as a reduction in antiviral CD4^+^ T cell functionality (specifically IL-2 production). Furthermore, these high antigen loads were shown to be important for sustaining the exhaustion phenotype and the level of antigen, not the strength of TCR stimulation, initiates the exhaustion phenotype [46,47,48]. Interestingly, it has also been suggested that the kinetics of antigen presentation, with NP antigens being expressed before GP antigens, and the inability of NP-recognizing T cells to withstand the increased antigen burden during LCMV Cl 13 infection both contribute to the impaired T cell response during chronic viral infection [14,49]. This may explain why NP396-recognizing CD8^+^ T cells, the primary cytotoxic lymphocyte directed against LCMV Arm, are rapidly diminished following LCMV Cl 13 infection [12,37,50,51]. 

### 5.2. Disruption of Lymphoid Organs

The physical disruption of lymphoid architecture is also responsible for the onset of viral persistence. As noted, the mutation in GP-1 of LCMV Cl 13 led to increased infectivity of macrophages and dendritic cells [7,52,53,54,55]. Work with LCMV strains that cause persistent infections showed that these infected cells could be targeted by cytotoxic lymphocytes, leading to a disruption of the lymphoid tissues and an impairment of the antigen-presentation process [56,57]. Further analysis showed that the lymphoid architectural cells, fibroblastic reticular cells, were also infected with LCMV Cl 13, which interrupted several lymphocyte processes that are regulated by these cells and led to the increased disruption of the lymphoid structure. A more recent study has also shown that LCMV Cl 13 triggers severe thymic depletion due to cytotoxic lymphocyte-mediated killing of infected cells in the thymus [58]. While the thymus tissue eventually recovers during chronic infection, the early disruption of this tissue contributed to an inability to replenish antiviral T cells early during the establishment of persistence. 

### 5.3. Inhibitory Receptors

The increased levels of inhibitory receptors are one of the more popular subtopics in the study of persistent viral infections as these represent potential therapeutic targets. Therapeutics that target inhibitory receptors discovered by the experimental use of LCMV Cl 13 have also been analyzed and confirmed with other persistent viral infections and in cancers (and vice versa). In this section, several key inhibitory receptors and their roles during LCMV Cl 13 infection are discussed. 

#### 5.3.1. PD-1/PD-L1

In 2006, a ground-breaking study identified PD-1, a known inhibitory receptor of TCR co-stimulation, as highly upregulated on exhausted CD8^+^ T cells during Cl 13 infection [17,59,60]. PD-1 binding to PD-L1/PD-L2 on target cells/antigen presenting cells disrupts TCR signaling and inhibits T cell proliferation [61]. It was observed that antibody-mediated disruption of PD-1 signaling via targeting of PD-L1 led to increased antiviral CD8^+^ T cell functionality and viral clearance independent of CD4^+^ T cell help [17]. Another study added to these findings to show that tissue expression of PD-L1 often correlated with PD-1 expression of exhausted CD8^+^ T cells in those tissues [62]. Also, viral persistence may result from PD-1^hi^ CD8^+^ T cell populations in specific tissues, including the bone marrow and liver. However, blockade of the PD-1/PD-L1 pathway does not completely restore CD8^+^ T cell functionality but may act on a specific subset of exhausted CD8^+^ T cells [17,63]. The levels of PD-1 were also shown to be high on exhausted CD4^+^ T cells during LCMV Cl 13 infection [38]. Nevertheless, it is unknown how important PD-1 on CD4^+^ T cells is to the exhaustion phenotype. Targeting PD-1 has since been applied to multiple areas of immunology for its role in repressing the immune response [64].

#### 5.3.2. LAG-3

Lymphocyte activation gene-3 (LAG-3) is a molecule expressed on activated CD4^+^ and CD8^+^ T cells that negatively regulates T cell function through its interactions with MHC-II [65,66,67]. LAG-3 is structurally similar to the CD4 molecule making it a competitor with CD4 for MHC-II binding. Since CD8^+^ T cells do not interact with MHC-II, LSECtin, which is expressed in the liver, has been suggested as a binding partner for LAG-3 [68,69,70]. LAG-3 can be strongly activated by IL-12, a pro-inflammatory molecule, and it is expressed on T cells in inflamed tissues as opposed to lymphoid tissues. LAG-3 was shown to be highly upregulated on exhausted CD8^+^ T cells during LCMV Cl 13 infection [18,62]. However, LAG-3 expression peaks early during infection and, unlike PD-1, seems to wane throughout the exhaustion phase [18]. LAG-3 expression slightly reduces the rate of CD8^+^ T cell division during LCMV infection, which may point to its function during exhaustion [71]. Antibody-mediated blockade of LAG-3 did not drastically increase the population of antiviral CD8^+^ T cells but did lead to a reduction of LCMV Cl 13 viral titers. Importantly, dual blockade of PD-1 and LAG-3 led to a significant increase in the functionality of antiviral CD8^+^ T cells during LCMV Cl 13 infection [62]. 

#### 5.3.3. Tim-3

Tim-3 is expressed on Th1 CD4^+^ T cells and cytotoxic CD8^+^ T cells [72]. Binding of Tim-3 with one of its ligands, galectin-9, induces cell death [73]. Another ligand, Ceacam-1, was identified for Tim-3, which is vital for the inhibitory function of Tim-3 and is important for the role of Tim-3 in exhaustion in a tumor setting [74]. Tim-3 regulates downstream TCR signaling, thus mediating its suppressive function [69,74,75]. During LCMV Cl 13 infection, Tim-3 was found to be co-expressed with PD-1 and was associated with a severe exhaustion state [76]. This has subsequently made Tim-3 a favorable marker for CD8^+^ T cells that have reached the advanced exhaustion stage as opposed to the memory-like phenotype (i.e., Tim-3^−^) [23,29]. Similar to other inhibitory molecules, Tim-3 blockade alone only slightly increased the function of antiviral CD8^+^ T cells and reduced LCMV titers in the liver by a minimal amount but not at all in the serum [76]. However, the dual blockade of Tim-3 and the PD-1/PD-L1 axis significantly increased antiviral CD8^+^ T cells and viral clearance. The role of Tim-3 on exhausted CD4^+^ T cells is currently unknown. 

#### 5.3.4. CD160

Interestingly, CD160, a binding partner of herpes virus entry mediator (HVEM), has been found to be a negative regulator of T cell activation and is upregulated on exhausted CD8^+^ T cells during LCMV Cl 13 infection [18,62,77,78]. However, the exact mechanism of CD160 signaling is unknown. Blocking of CD160 improved CD8^+^ T cell cytotoxicity and survival of PD-1^hi^ CD8^+^ T cells, in an in vitro experiment [62]. CD160 may only be expressed on a subset of exhausted CD8^+^ T cells late during infection making it a player in sustaining the persistent infection. 

#### 5.3.5. TIGIT

T cell immunoglobulin and immunoreceptor tyrosine-based inhibitory motif domain (TIGIT) is another immunoglobulin superfamily member that was found to be upregulated on exhausted CD8^+^ T cells during LCMV Cl 13 infection [79,80]. TIGIT competes with CD226 (a costimulatory molecule) for the ligand poliovirus receptor (PVR) and functions by disrupting downstream TCR signaling [69,81,82]. While in vivo antibody-mediated blockade against TIGIT alone did not restore antiviral function in CD8^+^ T cells, co-blockade with PD-1 synergistically reduced viral titers and increased the proportion of IFNγ^+^ CD8^+^ T cells [80]. 

#### 5.3.6. 2B4

As a CD2 receptor family member, 2B4 (CD244) has primarily been studied for its role in inhibition of NK cells [83,84]. Additionally, 2B4 is found on some CD8^+^ T cells and may function in a similar suppressor role; yet this T cell suppressive function is debated by some studies [83,85,86]. 2B4 was found to be upregulated on exhausted CD8^+^ T cells during LCMV Cl 13 infection and is mostly sustained during the infection [18,62]. The transfer of 2B4-deficient virus-specific CD8^+^ T cells during a persistent infection promotes sustained cell viability, which is not seen in the presence of 2B4 [16]. The blocking of 2B4 signaling did not enhance the cytotoxic ability of exhausted CD8^+^ T cells but did increase IFNγ production [62]. 2B4 appears to be biased towards exhausted CD8^+^ T cells rather than exhausted CD4^+^ T cells [38]. 

### 5.4. Immunoregulatory Cytokines

Several cytokines known to display immunoregulatory function during an immune response have also been shown to promote an immunosuppressive environment during LCMV Cl 13 infection. Several of these are discussed below. 

#### 5.4.1. IL-10

IL-10 is known for its suppressive functions on an array of immune cells [87]. During LCMV Cl 13 infection, IL-10-producing, virus-specific CD4^+^ T cells significantly increase in the spleen and the liver [36]. These cells were found early during infection but were diminished by 9 days post infection. Also, IL-10 producing DCs were increased during Cl 13 infection. Further analysis utilizing blockade of the IL-10 receptor or IL-10-deficient mice showed that IL-10 contributes to the establishment of a persistent infection [88]. This treatment enhanced antiviral CD8^+^ T cell responses, reduced PD-1-expressing CD8^+^ and CD4^+^ T cells and promoted viral clearance. IL-10 was shown to function in a distinct mechanism from PD-1/PD-L1-mediated suppression [89]. Importantly, this allowed a combinatory blockade of PD-L1 and IL-10 to increase the antiviral response against LCMV Cl 13 and promoted viral clearance. One of the major cell types identified to produce IL-10 during LCMV Cl 13 infection is virus-specific Th1 CD4^+^ T cells [90]. Thus, exhausted CD4^+^ T cells themselves appear to be reprogrammed, ultimately contributing to the exhaustion environment. 

#### 5.4.2. Type I IFN

Type I interferons (IFN-I) are well known for their role in antiviral responses [91]. While IFN-I was first discovered as an antiviral molecule that inhibits influenza virus replication in 1957 [92], its immune regulatory activities have also been revealed during virus infection [93]. The T cell protective function of IFN-I signaling has been well investigated during acute virus infection. For example, using the adoptive transfer of LCMV epitope-specific T cells, the expression of IFN-I receptor on CD8^+^ T cells was proven to be important for effector T cell expansion and memory formation [94]. The underlying mechanism for IFN-I was shown to be mediated by blockade of natural killer (NK) cell-mediated T cell lysis through the regulation of natural cytotoxicity triggering receptor 1 (NCR1) ligand on T cells [95]. However, during a chronic viral infection IFN-I signaling has been shown to negatively impact the adaptive immune response. IFN-I levels are elevated early during LCMV Cl 13 infection [96]. Similarly, IFN-I stimulated genes are upregulated and remain elevated in spleen tissue [97]. Blockade of the type I IFN receptor (IFNAR) leads to decreased PD-L1 expression on LCMV-infected DCs and on macrophages as well as a decrease in IL-10 levels in the serum early during infection [96,97]. Furthermore, blocking IFN-I signaling promotes control of viral persistence, which is due to an increase in virus-specific CD4^+^ T cells. IFN-I signaling is responsible for suppressing the formation of antiviral CD8^+^ T cells by antagonizing the TCF1-mediated generation of stem-like CD8^+^ T cells. IFNAR1 blockade increases the proportion of TCF1^+^ CXCR5^+^ Tim-3^−^ CD8^+^ T cells during LCMV Cl 13 infection [26,98]. 

Exhaustion of CD4^+^ T cells during LCMV Cl 13 infection has also been associated with chronic IFN-I signaling [38]. Even with low IFN-I levels later during LCMV Cl 13 infection, virus-specific CD4^+^ T cells appear to respond to IFN-I signals. In relation to other cells, IFN-I is implicated in the disruption of B cell generation during LCMV infection, which is defined as “B cell decimation.” This process involves the depletion of virus-specific B cells due to the generation of short-lived plasma cells [99]. This effect was independent of IFN-I signaling intrinsic to B cells. Instead, the process required a coordinated effect from DCs, T cells, and myeloid cells. Of note, IFN-I signaling also prevents the development of DCs during LCMV Cl 13 infection [100] where its signaling is dependent on STAT2 but independent of STAT1. The transient decrease in functional DC population will substantially disturb the antiviral host immunity required for controlling viral spread.

Finally, a recent study has shown that IFN-I signaling during LCMV Cl 13 infection imposes changes on liver cell metabolism, which downregulates antiviral T cell responses [101]. Signaling through IFNAR caused a break in the urea cycle, which is an important metabolic pathway in the liver, leading to an alteration of arginine and ornithine ratios in the serum. This change in metabolites was shown to impact the ability of virus-specific CD4^+^ and CD8^+^ T cells to respond to the LCMV infection but simultaneously protected the liver tissue from T cell-mediated damage. Overall, IFN-I appears to play a central role in the induction of exhaustion due to its potent immunoregulatory abilities during chronic infection. 

#### 5.4.3. TGF-β

TGF-β is a well-characterized cytokine known for its role in inhibiting immunopathology [102]. Virus-specific CD8^+^ T cells were shown to have increased TGF-β expression during LCMV Cl 13 infection [103]. In the absence of TGF-β signaling, virus-specific CD8^+^ T cells had reduced levels of the pro-apoptotic molecule Bcl-2-like protein 11 (Bim), which correlated with increased survival and functionality of both virus-specific CD4^+^ and CD8^+^ T cells during infection. Furthermore, TGF-β-deficient mice had reduced LCMV Cl 13 titers, which was dependent on CD8^+^ T cells and partially on CD4^+^ T cells. The observed effects on CD8^+^ T cells occurred through intrinsic TGF-β signaling and extrinsic effects of TGF-β on other cells. However, subsequent studies showed that antagonizing TGF-β signaling, as opposed to complete deletion, was only able to minimally increase virus-specific T cell responses and did not promote viral clearance [104,105]. These studies imply that TGF-β may be integral in contributing to the exhaustion state but not necessary to its continued maintenance. 

### 5.5. Regulation of Transcription and Epigenetic Modification

During T cell exhaustion, exhausted cells have been shown to acquire a distinct state of epigenetic and transcriptional changes that affect gene expression. These are distinct from effector and memory CD8^+^ T cells during acute viral infection [18,106,107,108]. Interestingly, changes in regulatory regions in exhausted CD8^+^ T cells were found to be typically associated with the activation of local genes, rather than the repression of those genes [106,107]. For instance, chromatin accessible regions related to the genes for inhibitory receptors PD-1 and Tim-3 were found to be open in exhausted cells [106]. Exhausted T cells appear to contain an open enhancer region for the *Pdcd1* (PD-1) locus, inaccessible in normal CD8^+^ T cells. Transcription factors commonly associated with exhausted T cells were also shown to localize to this enhancer region. Nevertheless, many accessible gene regions are shared between exhausted CD8^+^ T cells and normal CD8^+^ T cells, but the differences arise in the gene regulatory areas [107]. Interestingly, another study found that PD-1/PD-L1 blockade did not significantly change the epigenetic organization in exhausted CD8^+^ T cells [108]. Thus, PD-1 may regulate signaling and transcriptional events in cells, but it does not appear to regulate the epigenetic programming of exhausted T cells. Furthermore, PD-1/PD-L1 blockade failed to generate a lasting effector or memory phenotype from exhausted CD8^+^ T cells. This implies that changes in exhausted T cells are seemingly hard-wired in the genome and therapeutics may need to target upstream regulators of chromatin remodeling or specific transcriptional regulators that affect gene expression to alleviate the exhaustive state. 

Differential expression patterns of transcription factors in both exhausted CD8^+^ and CD4^+^ T cells have been observed [18,38]. These are often considered the regulatory mechanisms that influence the exhaustive state. In exhausted CD8^+^ T cells, regulatory changes were associated with not only an increase in inhibitory receptors but also changes in intracellular signaling pathways and changes in metabolic regulation [18]. Exhausted CD4^+^ T cells did show some of the conserved regulatory pathways of exhausted CD8^+^ T cells, but additional changes were seen in distinct transcription factors. Also, exhausted CD4^+^ T cell transcriptional patterns displayed a striking correlation with IFN-I signaling [38]. Several regulatory factors that have been identified as important during T cell exhaustion are discussed in this section.

#### 5.5.1. TOX

Thymocyte selection-associated high mobility group box protein TOX (TOX) has recently been identified as a critical transcriptional factor associated with the development of exhaustion [109,110,111]. TOX was shown to be required for the development of exhausted CD8^+^ T cells through its impact on modulating epigenetic and transcriptional regulatory features associated with the exhaustion state [110]. TOX was initially found to be induced by calcium signaling via calcineurin, which functions through the transcriptional regulator nuclear factor of activated T cells (NFAT) 2. TOX was shown to be required for the formation of stem-like exhausted CD8^+^ T cells and differentiates them from normal memory precursor CD8^+^ T cells [109,111]. Moreover, TOX promoted the survival of this subset of exhausted CD8^+^ T cells as well as terminally exhausted CD8^+^ T cells. Therefore, TOX may play an important role in the early differentiation into the exhaustion state but could also impart longevity on these cells. 

#### 5.5.2. NFAT

NFAT, a group of transcription factors, normally involved in T cell activation, has consistently been associated with the exhaustion state [18]. NFAT proteins interact with the Fos-Jun (activator protein 1, AP-1) transcription factors [112]. Modification of NFAT to prevent its partnering with AP-1 was shown to control aspects of T cell exhaustion through its binding to the regulatory regions of exhaustion-associated genes, like *Pcdc1* and *Havcr2* [113]. Interestingly, Fos, a binding partner of NFAT, was shown to be downregulated during LCMV Cl 13 infection, while NFAT was increased [18]. This gives substance to the role of unpartnered NFAT in regulating exhaustion. NFAT activity may also promote the establishment of the exhaustion phenotype through changes in active chromatin regions as well as transcriptional activation of genes [107]. NFAT is capable of inducing PD-1 expression. As a result, NFAT-deficient mice were shown to have fewer virus-specific CD8^+^ T cells expressing PD-1, Tim-3, and LAG-3 during LCMV infection [113,114]. Moreover, PD-1/PD-L1 blockade reduced the expression levels of targets for unpartnered NFAT [108]. 

#### 5.5.3. TCF1

TCF1 is important for the generation of memory and Tfh cells and is suggested to play a role in long-term T cell maintenance [26,115,116]. TCF1 was identified as a critical transcription factor in the generation of the memory-like subset of exhausted CD8^+^ T cells [20,23,25,26]. TCF1 is critical for maintaining this population during the exhaustion phase, which contributes to eventual viral clearance. TCF1-expressing exhausted CD8^+^ T cells express PD-1 but have lower levels of inhibitory receptors that are associated with more terminally exhausted cells (e.g., Tim-3) [26]. Moreover, these cells have enhanced proliferative capabilities [20]. TCF1 works in conjunction with Bcl6 to mediate this phenotype but was shown to repress Blimp-1, which antagonizes CXCR5 expression crucial for the function of these cells [25,26]. Later work showed that TCF1 functions early during the generation of the exhaustion state to prevent the formation of terminally differentiated effector cells [117]. PD-1 expression in this group of cells may protect them from alternative differentiation or deletion. Furthermore, this early regulation may facilitate the actions of TOX to provide stable epigenetic changes for the preservation of these memory-like cells [117]. Another transcription factor Forkhead box O1 (FOXO1), which can regulate TCF1 expression, has also been shown to be vital for sustaining the memory-like population of exhausted cells during LCMV Cl 13 infection [118]. Thus, TCF1 may be an essential regulator that positions T cells to differentiate towards the exhaustion state. While this seems to be a negative consequence, it may also prevent burn-out or host damage from over-activated T cells. 

#### 5.5.4. T-bet/Eomes

The transcription factor T-bet is often associated with its role in effector T cells. During LCMV Cl 13 infection, T-bet functions to repress exhaustion [79,119]. T-bet levels were lower in virus-specific CD8^+^ T cells following LCMV Cl 13 infection compared to LCMV Arm infection due to the higher antigen loads seen during LCMV Cl 13 infection [119]. In the absence of T-bet, LCMV Cl 13 titers are higher and antiviral CD8^+^ T cells are less functional. Furthermore, T-bet was shown to repress PD-1, LAG-3, and CD160 expression, and specifically repressed PD-1 expression at the transcriptional level. Therefore, T-bet may play a critical role in the maintenance of the effector function of exhausted CD8^+^ T cells. Alternatively, T-bet may promote an antiviral response but loses out to pro-exhaustion factors early during infection. 

Eomes is another transcription factor that is upregulated in exhausted CD8^+^ T cells. However, it is generally associated with effector T cells and generation of a memory CD8^+^ T cell phenotype [18,79,120]. Eomes was found to be upregulated during LCMV Cl 13 infection and its expression represented a more terminally differentiated population [121]. Furthermore, the Eomes-expressing cells appeared to be derived from the T-bet-expressing population but had enhanced proliferating capabilities. Thus, T-bet-expressing cells and Eomes-expressing cells may represent distinct populations of exhausted CD8^+^ T cells that both play roles in the antiviral response [119,121]. In fact, Eomes expression was increased in the memory-like population of exhausted CD8^+^ T cells expressing TCF1 [20]. These studies show the complexity of differential populations within the exhausted state as well as the importance of their maintenance for eventual viral control. 

#### 5.5.5. Blimp-1

Blimp-1 is a transcriptional repressor known to regulate cytokine expression in T lymphocytes as well as regulate B cell development [122]. Blimp-1 expression is increased in exhausted CD8^+^ T cells [18,62]. Deletion of Blimp-1 led to increased virus-specific CD8^+^ T cells and decreased PD-1, LAG-3, CD160, and 2B4 protein levels [62]. Like other transcriptional regulators the exhaustion-promoting function of Blimp-1 is necessary for eventual viral control, and Blimp-1-deficiency led to moderately increased viral levels. As noted, Blimp-1 antagonizes generation of the memory-like population of exhausted CD8^+^ T cells [25]. In another study, Blimp-1 was also found to promote the expression of IL-10 from Th1 cells due to constant antigen exposure [90]. Therefore, Blimp-1 may play important roles in the generation of effector-like exhausted CD8^+^ T cell populations that are required for intermediate viral control before the resolution phase but also in the generation of regulatory CD4^+^ T cells that contribute to the immunosuppressive environment. 

### 5.6. Other Markers

#### 5.6.1. Sphingosine Kinase 2

We have recently identified a cellular protein, sphingosine kinase 2 (SphK2), which is important for regulating T cell exhaustion, specifically through an intrinsic mechanism in CD4^+^ T cells [123]. SphK2 is an enzyme responsible for the generation of sphingosine 1-phosphate (S1P), a bioactive lipid metabolite, which regulates diverse cellular and disease conditions. While SphK2 has been associated with regulation of the replication process of viruses including influenza A virus, little is known regarding its function in host immunity to infection [124,125]. SphK2 as well as the activated, phosphorylated form of SphK2 were shown to increase in CD4^+^ T cells during LCMV Cl 13 infection, and deletion of SphK2 led to increased virus-specific T cell responses resulting in immunopathologic fatality of infected mice with nephrosis [123]. Thus, SphK2 negatively regulates CD4^+^ T cell functionality, which extrinsically contributes to the exhaustion of virus-specific CD8^+^ T cells. Use of a SphK2-selective inhibitor led to an increase in virus-specific, antiviral cytokine producing CD8^+^ and CD4^+^ T cells and consequent acceleration of viral clearance. The effects on viral resolution could even be observed if the inhibitor was given after the establishment of a chronic viral infection, which suggests that SphK2 is important for sustaining T cell exhaustion during the chronic stage of infection. Although the exact action of SphK2 on CD4^+^ T cells is unknown, significant changes in transcriptional regulation and cell cycle progression was observed in SphK2-deficient CD4^+^ T cells. Furthermore, SphK2-deficient CD4^+^ T cells were able to proliferate better in response to antigenic stimulation than SphK2-sufficient cells. Previous studies by others have shown the ability of SphK2 to repress histone deacetylase activity and regulate gene expression in the nucleus of cancer cells as well as regulate DNA synthesis [126,127]. Thus, SphK2 may play a role in mediating the exhaustion state of CD4^+^ T cells through epigenetic or transcriptional manners which warrants further investigation.

In addition, CD2AP was reported to regulate CD4^+^ T cell differentiation during chronic LCMV infection [41], but SphK2 deletion did not affect the expression of *Cd2ap* from the RNA-Seq analysis performed by us (not shown). This suggests that SphK2 functions in CD4^+^ T cells independently of CD2AP. 

#### 5.6.2. PSGL-1

A recent finding identified P-selectin glycoprotein ligand-1 (PSGL-1) as a novel regulator of CD8^+^ and CD4^+^ T cells exhaustion. PSGL-1 is normally involved in T cell motility; however, PSGL-1-deficient CD4^+^ and CD8^+^ T cells have reduced inhibitory receptor levels and increased antiviral functionality against LCMV Cl 13 [128]. PSGL-1 appears to function through the extracellular signal-related kinases (ERK) and protein kinase B (AKT) signaling pathways, which are known to regulate T cell responses. Upregulation of PSGL-1 promotes increased PD-1 levels and reduces TCR stimulation and IL-2 signaling. PSGL-1 was also found to be linked to transcriptional changes that promote CD8^+^ T cell exhaustion. In the absence of PSGL-1, the observed enhanced T cell functionality was linked to increased expression of the IL-7 and IL-2 receptors. Reduction of both of these signaling pathways are hallmarks for the exhaustion phenotype due to their roles in T cell proliferation and the memory response. Furthermore, several other inhibitory receptors (e.g., BTLA, CD160) were decreased on PSGL-1-deficient T cells, which may indicate that PSGL-1 is involved in upstream regulatory events that contribute to terminal exhaustion. Finally, CD4^+^ T cells were critical to the increased effector response of exhausted CD8^+^ T cells in the PSGL-1-deificient mice, indicating that PSGL-1 may play a role in the exhaustion of both CD4^+^ and CD8^+^ T cells [128]. 

#### 5.6.3. PTPN22

Protein tyrosine phosphatase non-receptor 22 (PTPN22) is traditionally associated with diverse autoimmune diseases as well as playing a role in T cells and innate immune cells [129]. Interestingly, during LCMV Cl 13 infection PTPN22-deficient mice showed reduced viral titers at 14dpi in several tissues and viral clearance in serum by 14dpi [130]. The absence of PTPN22 led to reduced IFN-I levels produced by the DCs and T cells of infected mice. These reduced IFN-I levels were correlated with reduced expression of the cAMP responsive element modulator, CREM, which is known to block IL-2 production. Overall, PTPN22-deficiency increased the amount and functionality of virus-specific CD4^+^ T cells, which appeared to be cell-extrinsic, leading to a better virus-specific CD8^+^ T cell response. These findings were corroborated by another group soon after, showing that PTPN22-deficient mice exhibited a reduced CD8^+^ T cell exhaustion phenotype, and improved virus-specific CD8^+^ and CD4^+^ T cell responses, which depended on T cell-extrinsic factors [131]. Therefore, PTPN22 can promote persistent LCMV infection by acting on CD4^+^ T cells, which prevents efficient help for the cytotoxic lymphocytes. It may be important to note that, in contrast to other molecules mentioned in this review, PTPN22 deficiency does not lead to harmful effects on the infected host through an increased immune response. This may be due to PTPN22’s extrinsic function. 

#### 5.6.4. PTPN2 

Recently, Tyrosine-protein phosphatase non-receptor type 2 (PTPN2) was identified as a negative regulator for generating the terminally exhausted (Tim-3^+^) CD8^+^ T cell population [132]. While not a transcriptional factor itself, PTPN2 is normally associated with several important signaling cascades [133]. In the absence of PTPN2, the Tim-3^+^ terminally exhausted population was found to increase during LCMV Cl 13 infection. This occurs due to increased IFN-I signaling. Thus, PTPN2 appears to work against the IFN-I-induced mechanism of instigating terminal exhaustion. 

## 6. Resolution of Chronic Viral Infection

While LCMV Cl 13 is able to last long term when adult mice are infected intravenously, the virus is ultimately cleared from mice due to the anti-viral immunity after 60–100 days of infection. Therefore, this model also provided an opportunity to investigate how the anti-viral immunity can be strengthened over time to eradicate viruses. For example, when CD4^+^ T cells were depleted, LCMV Cl 13 could establish life-long persistence, indicating that the presence of CD4^+^ T cells is important for the eventual clearance of the virus [12]. Several studies were conducted to reveal the mechanism for the termination of chronic LCMV Cl 13 infection.

IL-21 produced by CD4^+^ T cells during LCMV Cl 13 infection and signaling through the IL-21 receptor is crucial for maintaining antiviral CD8^+^ T cell responses and ensuring clearance of infection [134,135,136]. In the absence of IL-21, CD8^+^ T cells cannot control the infection and are deleted [134]. These studies also showed that IL-21^+^ CD4^+^ T cells were diminished in LCMV Cl 13 infection compared to LCMV Arm infection [135]. This may provide a potential explanation for the lessened antiviral capacity during LCMV Cl 13 infection; even though, the reduced IL-21 production is eventually able to promote viral clearance. Conversely, the switch to IL-21 producing CD4^+^ T cells may serve to promote sustained antiviral CD8^+^ T cell response [134]. Recently, this study was expanded to show that IL-21 produced by CD4^+^ T cells during LCMV Cl 13 infection promoted the formation of CX3CR1^+^ CD8^+^ T cells during the exhaustion phase, which are important for maintaining viral control [29]. IL-21 can also promote B cell responses, the development of Th17 CD4^+^ T cells, and Tfh CD4^+^ T cells, which may assist in the resolution of inflammation and the infection [137,138]. 

The IL-6 family of cytokines and signaling through the IL-6 family receptors are also vital for LCMV Cl 13 clearance. IL-6-deficient mice were shown to be incapable of controlling the viral infection [139]. Additionally, IL-6 was shown to be important for the generation of Tfh CD4^+^ T cells (through upregulation of *Bcl6*) and B cell responses later during LCMV Cl 13 infection. Follicular dendritic cells were a suggested source of this IL-6. In addition, IL-27, another IL-6 family member, was shown to play a critical role in LCMV Cl 13 viral control [140]. Overall, signaling through the shared IL-6 family receptor, gp130, was crucial for promoting an antiviral response and producing IL-21 during LCMV Cl 13 infection. IL-27 can also contribute to the early innate antiviral response through its actions on DCs, which contributes to increased IFN-I levels but also is required for eventual viral clearance [141]. IL-6 can promote B cell responses by mediating the production of IL-21 by CD4^+^ T cells and inhibit the formation of Tregs [142,143]. Recently, IL-27 has been identified to play an intrinsic role in promoting the survival of CXCR5^+^ CD8^+^ T cells, which were important for maintaining the antiviral T cell response during LCMV Cl 13 infection [20,25,98]. Thus, IL-27 could oppose IFN-I signaling during LCMV infection. 

Both IL-21 and IL-6 are important for generating Tfh CD4^+^ T cells that are selected for during LCMV Cl 13 infection [39,139,144]. Ultimately, these cells promote an adequate B cell and neutralizing antibody response that aids in the clearance of the infection [145,146,147]. Interestingly, chronic infection with LCMV Cl 13 virus was shown to instigate more robust germinal center B cell responses and antibody production than was capable during LCMV Arm infection [148,149]. This may be a result of the CD8^+^ T cell-dominated response during LCMV Arm infection. Despite diminished T cell responses during chronic viral infection, eventually protective B cell responses develop, and viral clearance is achieved. 

Taken together, these studies show that many arms of the adaptive immune response are critical for the eventual clearance of LCMV Cl 13. The mechanisms behind the regulation of these immune responses are complex and appear to be precisely coordinated. 

## 7. Conclusions and Perspectives

**Figure 1:**
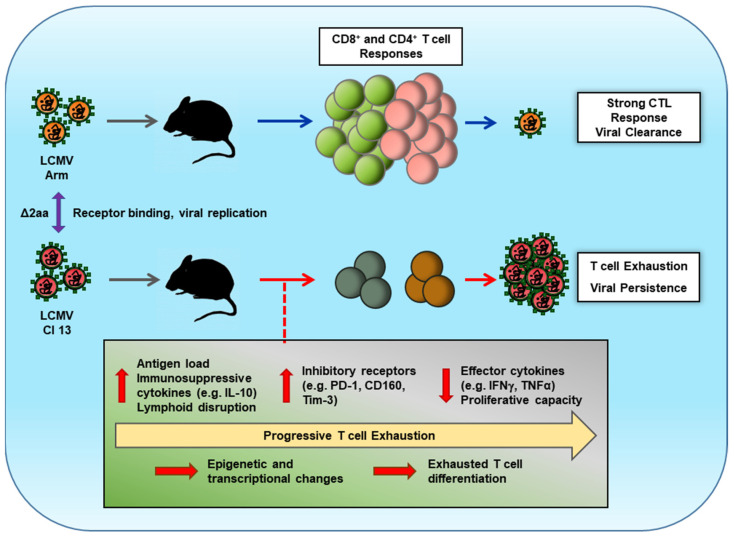
Infection of mice with differing LCMV strains leads to variable T cell responses impacting viral clearance. The prototype strain of LCMV, Armstrong 53b (Arm), readily infects mice. However, a strong host CD8^+^ T cell response and an ancillary CD4^+^ T cell response are able to clear the viral infection quickly. However, a variant strain of LCMV, Clone 13 (Cl 13), has two functional amino acid (aa) changes, which affect the virus’s ability to bind to its receptor and replicate in the host cell. These changes instigate a progressive exhaustion phenotype in both CD8^+^ and CD4^+^ T cells during LCMV Cl 13 infection. Studies have revealed that early, high antigen levels, the production of immunosuppressive cytokines, and disruption of lymphoid tissues promote epigenetic and transcriptional changes in T cells. These regulatory events lead to and occur in conjunction with increases in inhibitory receptor levels on exhausted T cells. Ultimately, exhausted T cells lose their ability to function and proliferate allowing the virus to persist in the host.

LCMV Cl 13 has been observed to cause drastic immunoregulatory events during infection, which functionally impairs the host immune response (Figure 1). These events are fascinating considering the similarity of LCMV Cl 13 to its variant strain LCMV Arm, which is rapidly cleared upon infection. Many programs contribute to instigating T cell exhaustion. Synthesis of immune regulatory molecules in the presence of high antigen burdens mediate changes in immune cells. The process is often associated with a change in gene transcriptional regulations that result in the increased expression of inhibitory receptors and other molecules to modulate the immune response. However, the immune protective signals can instill changes in antiviral T cells that burst and restore immune responses to promote clearance of the infection. Many of these concepts have been extended to chronic viral infections in humans as well as during cancer. Thus, the LCMV Cl 13 model has been a very influential contributor to our understanding of the immune response and continues to be a useful model for understanding virus-host defense interactions. Further research has the potential to lead to the development of new immune therapeutic maneuvers to cure viral diseases, as well as illnesses caused by immune dysregulation.

## Data Availability

Not applicable.
